# Numerical techniques to find optimal input parameters for achieving mean particles’ temperature and axial velocity in atmospheric plasma spray process

**DOI:** 10.1038/s41598-020-78424-w

**Published:** 2020-12-08

**Authors:** R. C. Batra, Unchalisa Taetragool

**Affiliations:** 1 Department of Biomedical Engineering and Mechanics, Virginia Polytechnic Institute and State University, Blacksburg, VA 24061 USA; 2 Department of Computer Engineering, King Mongkut’s University of Technology Thonburi, Bangkok, 10140 Thailand

**Keywords:** Engineering, Mathematics and computing

## Abstract

We numerically find values of four process input parameters, namely, the argon flow rate, the hydrogen flow rate, the powder feed rate, and the current, that yield the desired mean particles’ temperature and the mean particle velocity (collectively called mean particles’ characteristics, or MPCs) in an atmospheric plasma spray process just before the particles arrive at the substrate to be coated. Previous studies have shown that the coating quality depends upon the MPCs. The process is simulated by using the software, LAVA-P-3D, that provides MPCs close to their experimental values. Thus, numerical rather than physical experiments are conducted. We first use the design of experiments to characterize the sensitivity of the MPCs to process parameters. We then identify relationships between the significant input parameters and the MPCs by using two methods, namely, the least squares regression and the response surface methodology (RSM). Finally, we employ an optimization algorithm in conjunction with the weighted sum method to find optimum values of the process input variables to achieve desired values of the MPCs. The effects of weights assigned to the objective functions for the temperature and the velocity, and the difference in using the regression and the RSM model have been studied. It is found that these values of the process parameters provide MPCs within 5% of their desired values. This methodology is applicable to other coating processes and fabrication technologies such as hot forging, machining and casting.

## Introduction

Surface coatings are used to provide corrosion, wear and tarnishing resistance as well prolong electrical, optical, and/or thermal properties of substrate materials. Coatings allow depositing materials which are quite different from that of the substrate, and are categorized as thin (below a few micrometers in thickness) and thick (above 50 µm in thickness). Coating techniques include electroplating, chemical treatments, hot dip coating, chemical vapor deposition, physical vapor deposition (e.g., evaporation, puttering, ion plating), pulsed laser deposition, and thermal spray. These are briefly reviewed by Fauchais et al.^1^ who have listed in Table 2.1 of their chapter primary characteristics of the different methods. The methodology of finding appropriate input parameters for producing good quality coatings described in this paper is applicable to all these processes (and to several other manufacturing processes such as hot forging and machining) even though details are provided below only for a thermal spray process.

**Figure 1:**
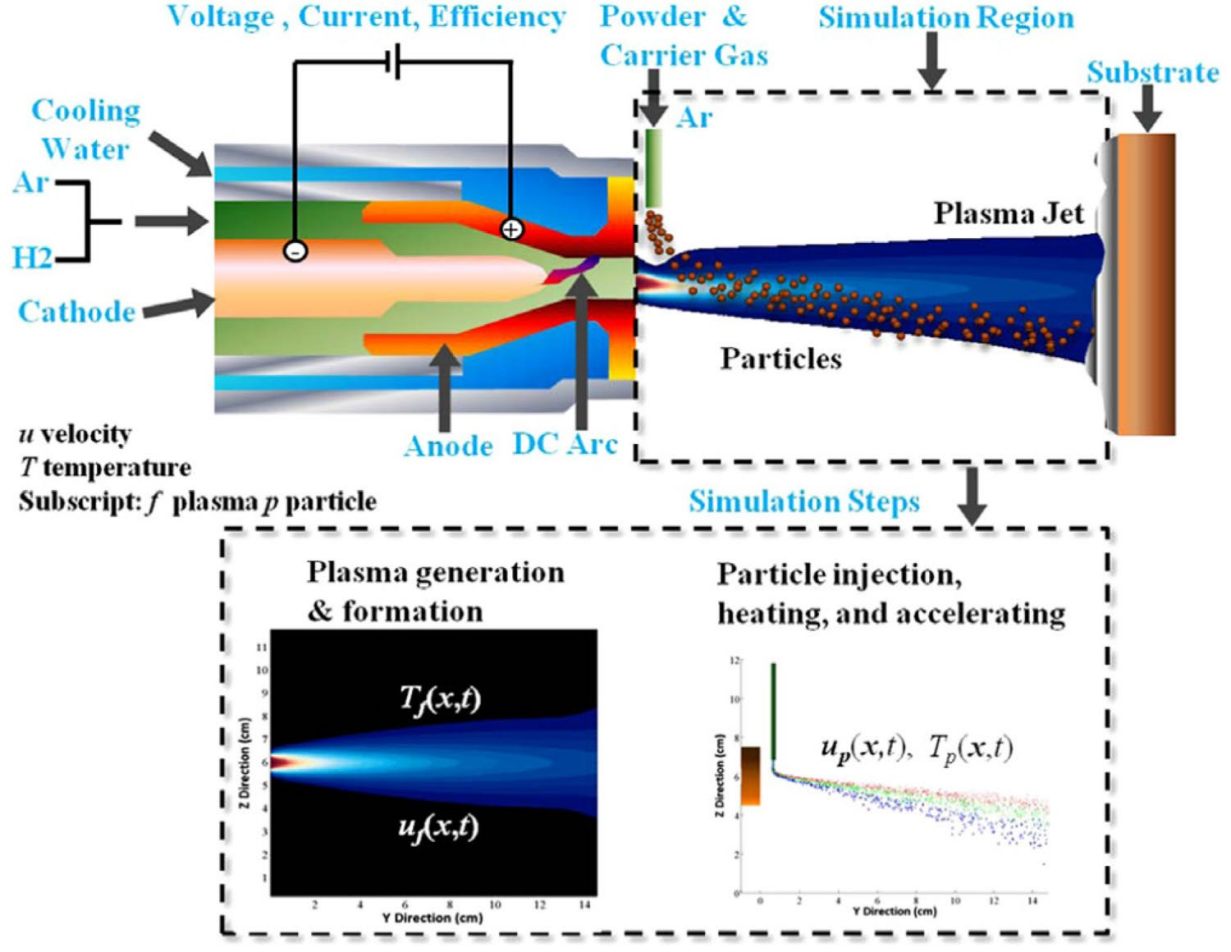
Schematics of an APSP, reproduced with permission from Shang et al.^2^ . Gases injected into the gas gun are decomposed into anions to form a plasma that exists the gun nozzle at a high velocity and elevated temperatures. The software, LAVA-P-3D, simulates the plasma flowing out of the nozzle as well as trajectories and temperatures of powder particles injected into the plasma from the powder port. The simulation domain is exhibited in the Fig. The plasma flow reaches steady state in about 4 ms when powder particles are randomly injected into it to achieve the prescribed mass/minute. The 1-cm wide observation window (not included in the Fig.) is located just before the substrate. In experiments as well as in simulations, temperatures and velocities of particles passing through the observation window are measured since their values affect the coating quality.

**Figure 2:**
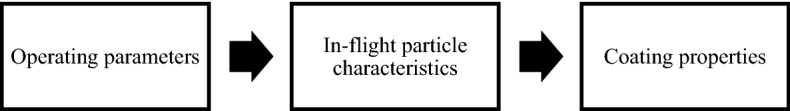
Three main aspects of an Atmospheric Plasma Spray Process.

An atmospheric plasma spray process (APSP) is a versatile thermal spray coating process that has been successfully used to coat components for gas turbines, airframes, engine and drive trains, and silicon chips. In an APSP (cf. Fig. 1) either metallic or ceramic (or their combination) powder particles injected into a very high temperature plasma jet formed by gases (usually a mixture of argon (Ar), hydrogen (H_2_), helium (He) and nitrogen (N_2_)) in an electric gun get heated while passing through the plasma, and are deposited on a prepared substrate. The coating quality is evaluated by the following three main properties: (i) physiochemical/thermochemical properties such as the hardness and the thermal conductivity, (ii) in-service performance that includes corrosion and wear resistance, and (iii) structural characteristics, e.g., the porosity, the thickness and the residual stresses. Variants of the APSP include vacuum low-pressure, inert, shrouded and controlled plasma spray. The main features of an APSP are summarized in Fig. 2.

Experimental and theoretical investigations^3,4^ have reported that the coating properties such as the coating hardness, wear resistance, roughness etc. (collectively called coating quality) depend on the in-flight mean particles’ characteristics (MPCs) when they strike the substrate and how the substrate was prepared. This work does not discuss the substrate preparation but focusses on the MPCs that represent the average values of particles’ states as they pass through an observation window, usually 1 cm wide, located just ahead of the substrate to be coated. The particles’ size, velocity and temperature, and whether or not they are partially/fully melted affect the coating quality. These characteristics can be adjusted through the plasma spray operating parameters.

An APSP has a large number of operating parameters such as the powder material, the powder port location and orientation, the voltage applied to the gun anode and cathode, their materials, the powder injection velocity, the range of diameters of powder particles, Ar and H_2_ (only these two are considered here) flow rates, the gun efficiency, and the substrate surface preparation and its location. It is an arduous task to conduct physical tests for quantifying how the coating quality depends upon each operating parameter. An economical alternative is to use a software and conduct numerical studies that also has the advantage of exploring a wide range of input parameters than that can be accessed experimentally. The software, LAVA-P, was developed by Ramshaw and Chang^5,6^ of the US Idaho National Engineering and Environmental Laboratory to analyze axisymmetric temperature and velocity fields in a plasma jet exiting the gas gun, and later extended by Wan et al.^7,8^ to predict particles’ characteristics. Xiong et al.^9^ generalized LAVA-P to LAVA-P-3D to consider effects of the carrier gas flowing with the powder particles on the plasma jet. Shang et al.^2^ incorporated in LAVA-P-3D effects of turbulent modulation. The software does not simulate plasma formation in the gas gun; an interested reader may consult Ref.^10^ and articles cited therein for modeling the plasma generation. However, it simulates the plasma flow as a mixture of various gases and ionization products exiting the gas gun, chemical reactions amongst plasma constituents, injection of powder particles into the plasma, trajectories of particles as they traverse through the plasma, heating of particles due to heat transfer between them and the plasma, and their melting. Effects of gases injected into the gas gun, the power input and the gun efficiency are accounted for by ensuring that the mass flow rate of the plasma ejecting out of the gas gun equals their input values. Shang et al.^2^ have summarized in Sects. 2, 3 and 4 of their paper the governing equations (i.e., the conservation of mass of each specie, the total mass, the linear momentum, and the total energy) for the plasma and particles’ heating and motion by using a lumped heat capacitance model, assumptions made in developing the mathematical model, the choice of the computational domain, the initial and the boundary conditions, and the numerical techniques used to solve these equations. They first compared the velocity and the temperature distributions in the plasma with the experimental results and then particles’ characteristics with experimental findings of other investigators. They found that the asymmetry introduced by the carrier gas does not significantly affect computed particles’ characteristics, and the computed velocity and temperature profiles matched well with the corresponding experimental results. Xiong et al.^9^ have also shown that the simulation results from LAVA-P-3D for the MPCs differ by less than 10% from their experimentally measured values. It has thus been established that the software predicted MPCs agree well with those observed experimentally. Here we use the software LAVA-P-3D to optimize input parameters for attaining desired values of MPCs.

Effects of process parameters on the coating quality are inter-related. A powder particle’s trajectory is influenced by its injection velocity, its diameter, and the plasma flow which is determined by, among other parameters, the Ar and the H_2_ flow rates. Thus, interactions among effects of different parameters must be considered to optimize the MPCs. Whereas results of physical experiments have previously been used to optimize the process parameters, we employ here results of simulations with the software, LAVA-P-3D. The data from physical experiments account for uncertainties in various variables but the range over which they can be changed is limited by the available facilities. Numerical simulations may not consider all of the physics of the problem and effects of sudden changes in the input parameters (sometimes called noise variables), but allow for studying an extensive range of the input parameters when most of the process relevant physics is included in the mathematical model used to develop the software. As noted above, LAVA-P-3D’s predictions of the MPCs are close to the experimental observations.

Heimann^11^ has reviewed a large body of literature on the optimization of the plasma-sprayed coatings' properties and performance using the design of experiments (DoE) and the artificial intelligence methods. Statistical design of experiments such as the factorial design, the Taguchi method and the response surface methodology (RSM) have been widely used to identify parameters that significantly influence the coating performance. The artificial neural network (ANN) has been used to optimize values of input parameters for producing a coating of desired quality.

In Table 1 we have listed key features of several studies on the APSP that have used the DoE to delineate the coating characteristics. Two main techniques employed are the factorial design and the RSM. The fractional factorial design which uses fewer number of simulations than those in the full factorial design has also been employed. The RSM has been categorized into the Box-Behnken design and the central composite design.

**Table 1:** Examples of the DoE applied to an APSP.

Technique	Powder material	Input: Operating parameters	Output: Characteristics/Properties of interest	Reference
Full factorial design	TiO_2_	Plasma power, powder feed rate, stand-off distance	Porosity, micro-hardness, surface roughness, wear rate	^12^
Fractional factorial design	MgAl_2_O_2_	Ar flow rate, current, spray distance, powder feed rate	Hardness, porosity	^13^
	Fly ash	Primary gas pressure, carrier gas pressure, powder feed rate, plasma power	Thickness, surface roughness, micro-hardness	^14^
	Al_2_O_3_–SiO_2_	Gun current, Ar flow rate, H_2_ flow rate, carrier gas flow rate, stand-off distance, substrate temperature	Thickness, porosity	^15^
Box-Behnken design	ZrO_2_–Y_2_O_3_	Spray layers, voltage, arc current, travel speed, spray stand-off, powder feed rate, carrier gas, primary gas flow rate	Profile hardness	^16^
	NiCrAlY	Aluminum (Al) and nickel (Ni) content of pack powder, process temperature	Thickness, Al/Ni ratio of coatings	^17^
	La_2_Ce_2_O_7_	Ar flow rate, H_2_ flow rate, arc current	MPCs, microstructure, hardness and fracture toughness	^18^
Central composite design	ZrO_2_	Power, stand-off distance, powder feed rate	Porosity, micro-hardness	^19^
	Al_2_O_3_	Power, stand-off distance, powder feed rate	Porosity	^20^

As listed in Table 1, Forghani et al.^12^ used the full factorial design to show that the input power and the powder feed rate (PFR) significantly influence the coating porosity, the micro-hardness, the surface roughness, and the wear rate. The stand-off distance was found to only affect the surface roughness, and the interaction between the plasma power and the PFR the coating porosity. Karthikeyan et al.^19^ estimated the coating porosity and micro-hardness by using the central composite design by considering the input power, the PFR, and the standoff distance as experimental factors. Pierlot et al.^21^ have reviewed several works and reported that the DoE is typically applied to the APSP to optimize the coating properties.

An artificial neural network (ANN) has been extensively utilized to analyze in-flight particle characteristics in an APSP and delineate complex non-linear relationships between the input process parameters and the MPCs. Experimental results were used to train the ANN and compare predictions from it with experimental findings. The arc current intensity, the Ar flow rate, and the H_2_ flow rate were generally used as input factors. Some works used a total plasma gas flow rate (i.e., the sum of Ar and H_2_ gas flow rates) and H_2_ content (i.e., the ratio of H_2_ flow rate to Ar flow rate) instead of the individual Ar and H_2_ flow rates. In Table 2 we have listed examples of the ANN applied to the APSP using experimental data. The ANN predictions were within 10% of experimental observations.

**Table 2:** Examples of an ANN applied to an APSP.

Spray Material	Input: Operating parameters	Output: Characteristics/Properties of interest	Reference
Al_2_O_3_–TiO_2_	Arc current, Ar flow rate, H_2_ flow rate, Ar carrier gas flow rate, injector diameter, injector stand-off distance	MPCs, mean particles’ diameter	^22^
Al_2_O_3_–TiO_2_	Arc current intensity, total plasma gas flow, H_2_ content	MPCs, porosity, deposition yield	^23^
Al_2_O_3_–TiO_2_	Arc current intensity, total plasma gas flow, H_2_ content	MPCs, mean particles’ diameter	^24^
Al_2_O_3_	Arc current intensity, Ar flow rate, H_2_ flow rate, Ar carrier gas flow rate, injector stand-off distance, injector diameter	MPCs, mean particles’ diameter	^25^

It is clear from works listed in Table 2 that the exploration of input parameters on the MPCs is of significant interest.

Three steps involved in finding optimal values of process parameters are screening, identifying relationships, and optimization. In the screening process, we use the factorial experiment to identify parameters that noticeably impact outputs of interest. Subsequently, we express the MPCs as quadratic functions of the significant input parameters by using the least squares method and the RSM. These relationships are then used in an optimization algorithm as an objective function to find values of the input parameters to achieve desired values of MPCs. Basically the optimization algorithm solves two nonlinear algebraic equations for the four significant input parameters. Finally, we check if the so found values of the optimum input parameters when used in LAVA-P-3D give the desired MPCs.

## Methodology

### Selection of input parameters

Among the several process parameters listed in the Introduction, the following four operating factors, namely, the current, the powder feed rate (PFR), the Ar flow rate and the H_2_ flow rate are believed to dominantly affect the MPCs. Even though the voltage in the gas gun fluctuates and plays a noticeable role, these fluctuations depend upon, among other factors, the Ar and the H_2_ flow rates and materials for the anode and the cathode, and are difficult to numerically simulate since no mathematical expression is available for them. The importance, if any, of the interactions among the four process parameters has not been characterized heretofore. The other operating parameters are kept fixed at the following values: Voltage = 63.7 V; gun efficiency = 54.9%; ambient gas, temperature and pressure = air, 300 K, 85.5 kPa, respectively; spray distance = 12.5 cm; nozzle radius = 4 mm; torch radius = 52 mm.

### Powder particles

**Figure 3:**
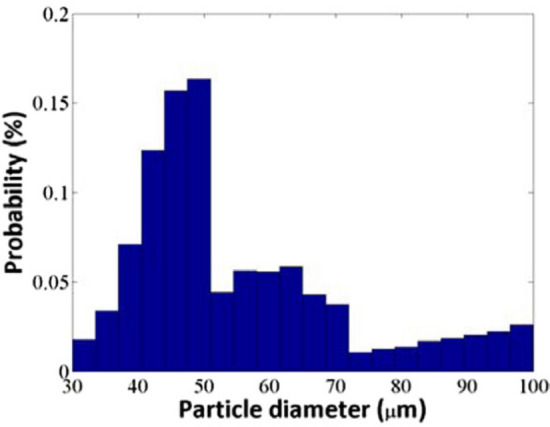
Histogram of powder particles’ distribution used in experiments by Wan et al.^3^ and in the current simulations.  Figure reproduced with permission from Shang et al.^2^.

Parameters for the ZrO_2_ powder particles are: mass density = 5.89 g/cm^3^, particle size = 30–100 μm, mean particle diameter = 58 μm, injection speed = 14.5 m/s, melting temperature = 2950 K, thermal conductivity = 2.0 W/(m K), heat capacity = 580 J/(kg K). These powder particles were used in experiments by Wan et al.^3^ who gave the probability of particles’ diameter exhibited in Fig. 3.

### Computation of mean particles characteristics

The software LAVA-P-3D is used to find the MPCs at any instant of time within the 1-cm wide window located just before the substrate. The plasma exiting the gun nozzle at time t = 0 typically reaches a steady state at t = 4 ms when powder particles from the powder port are injected into the plasma at random velocities to achieve the prescribed PFR. The software finds the drag force between a particle and the plasma, determines heat exchange with the plasma and computes particle’ trajectory and temperature.

### Screening of parameters using the factorial design

To identify input parameters that greatly affect MPCs, each input parameter (or factor) is assigned discrete values called “levels”. For two factors *A* and *B* having levels *a* and *b,* respectively, all *ab* combinations are included in the factorial experiment. The effect of a factor is called a main effect while that of the combination an interaction effect. The significant main and interaction effects are determined by using the analysis of variance (ANOVA).

To illustrate concepts, we consider three factors *A*, *B* and *C* with 5 levels each for which a factorial experiment is represented by Eq. 1.

1 $$Y_{ijk} = \mu + A_{i} + B_{j} + C_{k} + \left( {A*B} \right)_{ij} + \left( {A*C} \right)_{ik} + \left( {B*C} \right)_{jk} + \varepsilon_{ijk} ,\quad i, \, j, \, k = { 1},{ 2},{ 3},{ 4},{ 5}$$

where $$Y_{ijk}$$ denotes the response from the ith level ($$A_{i} )$$ of factor $$A$$, the jth level (B_j_) of factor $$B$$, and the kth level (C_k_) of factor $$C;$$ $$\mu$$ equals the overall mean effect; $$\left( {A*B} \right)_{ij}$$ the effect of the interaction between factors $$A_{i}$$ and $$B_{j} ;$$ and $$\varepsilon_{ijk}$$ a random error component.

The significant main and interaction effects are determined by deviations from the overall mean. The equality of effects states that the effect from each level of the factor is the same. Testing hypotheses about the equality of effects can be stated as follows.

2 $$H_{0} :A_{1} = A_{2} = \cdots = A_{4} = 0;H_{1} : {\text{at least one }}A_{i} \ne 0$$

3 $$H_{0} :\left( {A*B} \right)_{ij} = 0\,\, {\text{for all i, j;}}\, H_{1} : {\text{at least one }}\left( {A*B} \right)_{ij} \ne 0$$

**Figure 4:**
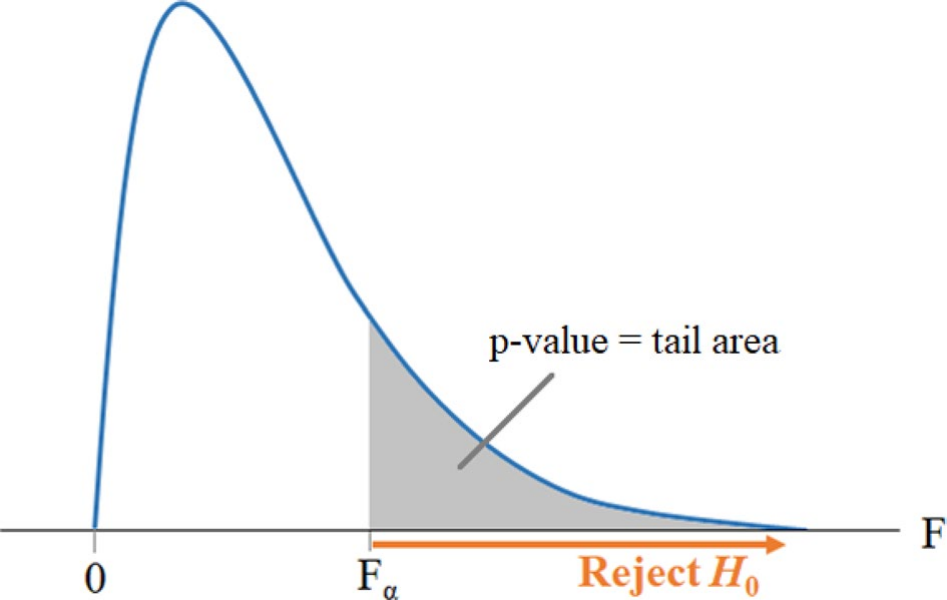
F-Distribution.

$H_{0}$ is called the “null hypothesis” and $H_{1}$ the “alternative hypothesis”. The hypotheses are tested by the ANOVA described in the Table included in the supplementary material for three factors A, B and C with four levels a, b, c, and d, respectively. The F- statistic equals the ratio of the variation between sample means and the variation within the samples. The upper tail of the F distribution found from the statistical Tables is used to test the hypotheses as shown in Fig. 4.The null hypothesis, $H_{0}$, is rejected if the F-value of the factor is in the region that is larger than $F_{\alpha }$ in Fig. 4.

### Regression analysis (RA)

Even though we use ANOVA to identify significant factors and interactions amongst them, for the RA and the RSM we use complete quadratic functional relationship between the mean particles’ temperature or velocity response, *y*, and four factors listed as $x_{1} ,x_{2} ,x_{3} ,x_{4} .$ The coefficients $\alpha$ in Eq. (4) are estimated by the RA and the RSM.

4 $$\begin{aligned} y & = \alpha_{0} + \alpha_{1} x_{1} + \alpha_{2} x_{2} + \alpha_{3} x_{3} + \alpha_{4} x_{4} + \alpha_{12} \left( {x_{1} *x_{2} } \right) + \alpha_{13} \left( {x_{1} *x_{3} } \right) + \alpha_{14} \left( {x_{1} *x_{4} } \right) + \alpha_{23} \left( {x_{2} *x_{3} } \right) \\ & \quad + \alpha_{24} \left( {x_{2} *x_{4} } \right) + \alpha_{34} \left( {x_{3} *x_{4} } \right) + \alpha_{11} x_{1}^{2} + \alpha_{22} x_{2}^{2} + \alpha_{33} x_{3}^{2} + \alpha_{44} x_{4}^{2} \\ \end{aligned}$$

For 5 levels of each factor, we get 5 sets of Eq. (4) which we write in the matrix form as

5 $$y = \left( {\begin{array}{*{20}c} {y_{1} } \\ {y_{2} } \\ \vdots \\ {y_{5} } \\ \end{array} } \right)\quad X = \left( {\begin{array}{*{20}c} 1 & {x_{1,1} } & {x_{1,2} } & \cdots & {x_{1,14} } \\ 1 & {x_{2,1} } & {x_{2,2} } & \cdots & {x_{2,14} } \\ \vdots & \vdots & \vdots & {} & \vdots \\ 1 & {x_{5,1} } & {x_{5,2} } & \cdots & {x_{5,14} } \\ \end{array} } \right)\quad \alpha = \left( {\begin{array}{*{20}c} { \alpha_{0} } \\ { \alpha_{1} } \\ { \alpha_{2} } \\ \vdots \\ { \alpha_{14} } \\ \end{array} } \right)$$

Thus

6 $$\alpha = X^{\prime}y$$

where $X^{\prime}$ is the inverse of the matrix $X$.

Each factor is normalized to vary between -1 and 1. We perform 625 numerical experiments and use the least squares method to find $\alpha$’s.

### Response surface methodology (RSM)

We used the Box-Behnken design^26^, which is an RSM, in our work. The Box–Behnken design requires three levels, -1, 0, and 1 (called “coded values”, respectively, for the low, the intermediate, and the high levels) of each factor to fit the quadratic relation. Considering Eq. (4), coefficients $\alpha_{0} , \alpha_{i} , \alpha_{ij}$ can be estimated by Eqs. (7a-d) listed below.

7a-d $$\begin{aligned} \alpha_{0} & = \overline{y}_{0} ;\,\alpha_{i} = A\mathop \sum \limits_{m = 1}^{n} x_{im} y_{m} ;\,\alpha_{ii} = B\mathop \sum \limits_{m = 1}^{n} x_{im}^{2} y_{m} + C\mathop \sum \limits_{l = 1}^{k} \mathop \sum \limits_{m = 1}^{n} x_{lm}^{2} y_{m} - \frac{{\overline{y}_{0} }}{s} \\ \alpha_{ij} & = D\mathop \sum \limits_{m = 1}^{n} x_{im} x_{jm} y_{m} \\ \end{aligned}$$

Following^26^, we set $A = 1/12, B = 1/8, C = - 1/48, D = 1/4,$ and $s = 2.$ $\overline{y}_{0}$ is the average value of the response when all input values are at the zero level, $y_{m}$ is the value of the response from the numerical experiments, while $x_{im}$ equals −1, 0, 1.

We analyze the Box–Behnken design with the four input parameters by using the statistical analysis software JMP and perform 27 numerical experiments listed in a Table in the supplemental material by using the software LAVA-P-3D.

### Input parameters for desired MPCs

The desired values of the MPCs equal the left hand sides of equations for the mean particles’ temperature and the mean particles’ velocity deduced by the RA and the RSM. These two nonlinear algebraic equations are simultaneously solved for the four input parameters by using an optimization algorithm, i.e., by minimizing the error between the values computed using the right-hand sides of these equations and the desired values, $T_{desired}$ and $V_{desired}$, of the mean particles’ temperature and velocity, respectively. Said differently, we find the four input parameters so that the error defined below by Eq. (8) is the minimum where *Tc* and *Vc* are, respectively, computed from Eq. 6 and the RSM, and 0 $\le w \le$ 1 is a weight function that determines importance assigned to the $T_{desired}$ and $V_{desired}$.

8 $$Error = \sqrt {w\left( {\frac{{T_{desired} - T_{c} }}{{T_{c} }}} \right)^{2} + \left( {1 - w} \right)\left( {\frac{{V_{desired} - V_{c} }}{{V_{c} }}} \right)^{2} }$$

It is clear that a solution of Eq. (8) depends upon the choice of *w*. The set of solutions obtained for different values of *w* is called the Paretto family. Approaches such as data mining and goal programming have also been employed to solve such problems^7^.

We minimize the error (Eq. 8) by using two optimization algorithms, namely, the NeSS (nest site selection) and the GA (genetic algorithms), respectively, described in Refs.^27^ and^28^.

## Results

### Screening of parameters using the factorial design

We consider the following five levels for the four factors: Ar flow rate (slm) = 30, 40, 50, 60 and 70; H_2_ flow rate (slm) = 3, 6, 9, 12 and 15; Current (Cur, A) = 300, 375, 450, 525, 600; PFR (g/s) = 0.2, 0.3, 0.4, 0.5, 0.6. Thus, ${5}^{4}$ = 625 runs required for the factorial experiment were simulated using LAVA-P-3D. The MPCs were used for the analysis of variance using the statistical software, JMP, developed by the SAS Institute. Values of the ANOVA variables are listed in Table 3.

**Table 3:** Analysis of Variance (ANOVA) for MPCs. The *p*-value less than 0.05 implies that the variable is significant.

Source	DF	Velocity			Temperature		
		Sum of Squares	F Ratio	*p*-value	Sum of Squares	F Ratio	p-value
Ar	4	33,865,492	2613.0	< .0001	212,641.8	5855.0	< .0001
H_2_	4	1,384,155	106.8	< .0001	3905.36	107.5	< .0001
Current	4	12,528,811	966.7	< .0001	118,688.3	3268.0	< .0001
PFR	4	8461	0.6529	0.6251	117.88	3.246	0.0121
Ar*H_2_	16	877,894	16.93	< .0001	2198.02	15.13	< .0001
Ar*Current	16	3,822,953	73.74	< .0001	2781.96	19.15	< .0001
Ar*PFR	16	8407	0.1622	0.9999	56.85	0.3913	0.9844
H_2_*Current	16	187,873	3.624	< .0001	7839.76	53.97	< .0001
H_2_*PFR	16	4360	0.0841	1.0000	55.29	0.3806	0.9866
Current*PFR	16	2,293,330	44.24	< .0001	15,443.91	106.3	< .0001

It is evident from the values listed in Table 3 that the significant main and interaction effects for the mean particles’ velocity are the same as those for their mean temperature, except that the PFR is also significant for the temperature. Therefore, all four factors and interactions Ar*H2, Ar*Cur, H2*Cur, and PFR*Cur should be used to find optimum values of the four input variables.

### Regression analysis (RA)

Using results of 625 numerical simulations and Eq. (4), we found the following expressions for the MPCs.

9 $$\begin{aligned} temp & = 2902.83 - 327.5 Ar + 196.0 Cur + 3.76 H_{2} - 4.175 PFR \\ & \quad + 39.72 Ar^{2} + 32.59 Cur^{2} - 112.26 H_{2}^{2} + 4.595 PFR^{2} + 10.95 \left( {Ar*Cur} \right) \\ & \quad + 53.63 \left( {Ar*H_{2} } \right) - 0.3731 \left( {Ar*PFR} \right) + 8.834 \left( {Cur*H_{2} } \right) \\ & \quad + 70.78 \left( {Cur*PFR} \right) - 1.628 \left( {H_{2} *PFR} \right) \\ \end{aligned}$$

10 $$\begin{aligned} vel & = 155.87 + 19.23 Cur - 0.3016 H_{2} + 0.3383 PFR + 26.02 Ar \\ & \quad - 0.3743 Ar^{2} + 0.8928 Cur^{2} - 5.228 H_{2}^{2} + 0.8157 PFR^{2} + 3.083 \left( {Ar*Cur} \right) \\ & \quad - 1.619 \left( {Ar*H_{2} } \right) + 0.2714 \left( {Ar*PFR} \right) + 6.617 \left( {Cur*H_{2} } \right) \\ & \quad + 5.449\left( {Cur*PFR} \right) + 0.1157 \left( {H_{2} *PFR} \right) \\ \end{aligned}$$

In Eqs. 9 and 10, the higher the coefficient of a parameter, the stronger the effect it has on the output. Thus, in Eq. 9, the Ar flow rate has the greatest influence on the particles’ mean temperature and velocity; the negative and positive signs of the coefficient indicate the direction of the effect. That is, increasing the Ar flow rate decreases the mean particles’ temperature but increases the velocity.

### Response surface methodology (RSM)

The MPCs from the 27 simulations using the RSM have the following expressions.

11 $$\begin{aligned} temp & = 2902.67 - 290.9 Ar + 154.8 Cur + 13.58 H_{2} + 20.43 PFR \\ & \quad + 49.46 Ar^{2} + 9.528 Cur^{2} - 96.51 H_{2}^{2} + 27.62 PFR^{2} + 3.077 \left( {Ar*Cur} \right) \\ & \quad + 89.74 \left( {Ar*H_{2} } \right) + 14.71 \left( {Ar*PFR} \right) + 45.44 \left( {Cur*H_{2} } \right) \\ & \quad + 33.32 \left( {Cur*PFR} \right) - 14.01 \left( {H_{2} *PFR} \right) \\ \end{aligned}$$

12 $$\begin{aligned} vel & = 155.063 + 26.22 Ar + 17.90 Cur - 0.2738 H_{2} + 1.016 PFR \\ & \quad + 1.448 Ar^{2} + 0.3325 Cur^{2} - 3.767 H_{2}^{2} + 2.125 PFR^{2} \\ & \quad + 5.541 \left( {Ar*Cur} \right) - 1.483 \left( {Ar*H_{2} } \right) + 0.6637 \left( {Ar*PFR} \right) \\ & \quad + 7.095 \left( {Cur*H_{2} } \right) + 6.547 \left( {Cur*PFR} \right) + 1.538 \left( {H_{2} *PFR} \right) \\ \end{aligned}$$

The different (625 vs. 27) number of experiments used in the RA and the RSM give different values of coefficients of identical terms in Eqs. 9 and 11 as well as in Eqs. 10 and 12.

**Figure 5:**
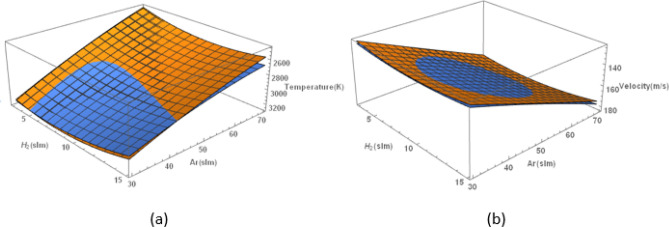
Plot of particles’ mean (**a**) temperature and (**b**) velocity versus the Ar and the H_2_ flow rates. The orange [blue] surface represents results from the RA [RSM]. The two surfaces are close to each other. For the Ar and the H_2_ flow rates lying on the curve of intersection of the two surfaces, the RA and the RSM give identical values of the MPCs.

Figure 5 illustrates plots of surfaces represented by the equations vs. the Ar and the H_2_ flow rates for the $\mathrm{current}=450\mathrm{ A}$ and the $\mathrm{PFR}=24\mathrm{ g}/\mathrm{min}$. The orange [blue] surface is for results from the RA [RSM]. It is clear that the two approaches provide qualitatively similar results for the MPCs.

### Input parameters for desired MPCs

For T_desired_ = 3000 K, V_desired_ = 160 m/s, and *w* = 0.5 values of the four input parameters found using the methodology described above are listed in Table 4.

**Table 4:** Optimal input parameters from the NeSS and the GA for the desired MPCs (3000 K, 160 m/s).

Method	Algorithm	optimal solution									Statistics of $\%{\text{Error}}_{{ {\text{obj}}}}$		
		Ar	H_2_	Current	PFR	$T$	$V$	${\%} {\text{ Error}}_{ T}$	${\%} {\text{Error }}_{V}$	${\%} {\text{Error}}_{{ {\text{obj}}}}$	Mean	Max	Min
RA	NeSS	47.94	9.20	484.5	0.6	2947	167.8	1.76	4.86	3.31	4.39	6.95	3.31
	GA	47.37	11.63	492.6	0.579	2967	169.7	1.10	6.07	3.58	4.83	7.19	3.58
RSM	NeSS	44.88	14.59	502.3	0.599	2997	169.0	0.12	5.62	2.87	4.59	7.00	2.87
	GA	45.16	14.36	500.4	0.596	2991	167.6	0.30	4.77	2.54	4.59	6.14	2.54

For the RA, the two optimization techniques provide close values for the Ar flow rate, the current and the PFR but not for the H_2_ flow rate. Values of mean particles temperature, *T*, and the mean particles’ velocity, *V*, listed in columns 7 and 9 of Table 4 and those derived by using the respective equations for them with values of the input parameters listed in columns 3, 4, 5 and 6 of Table 4 have errors of about 5–6% for *V* and 1–2% for *T*. Unlike for the RA, values of the four input parameters found using the NeSS and the GA algorithms are nearly the same for the RSM.

These exercises suggest that one should use the RSM for deriving approximate models of the APSP to express the MPCs as a function of the input parameters.

#### Effect of the weighting factor in Eq. (8)

**Figure 6:**
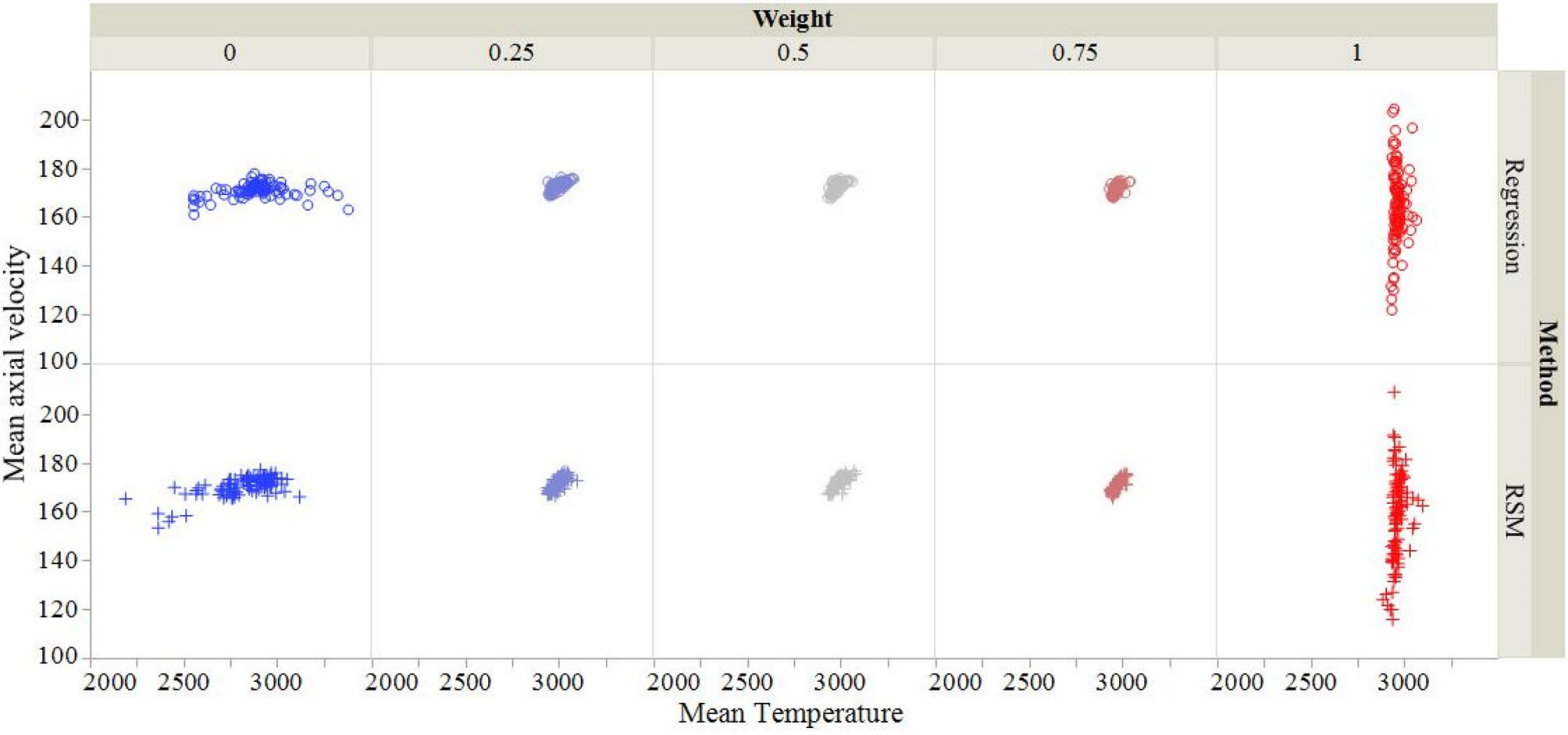
For *w* = 0, 0.25, 0.5, 0.75 and 1, distributions of the 100 optimal solutions from the RA and the RSM models. It is clear that for *w* = 0 (1), the mean axial velocity (mean temperature) is essentially unchanged for the 100 solutions. For w = 0.25, 0.5 and 0.75, the solutions are clustered together.

The solution from an optimization algorithm invariably depends upon initial estimates of the solution. For *w* = 0, 0.25, 0.5, 0.75 and 1 in Eq. (8), we have listed in Table 5 the best optimal solutions from 100 initial estimates and the RA and the RSM models. For *w* $\ne$ 0, except for the H_2_ flow rate, the other three input factors vary by a small amount for the remaining four values of *w*. It is transparent from plots of the MPCs exhibited in Fig. 6 that the solutions are clustered together for *w* = 0.25, 0.5 and 0.75. It suggests that one can assign any value to *w* between 0.25 and 0.75 without materially affecting values of the input parameters. However, values of the H_2_ flow rate depend upon *w*.

**Table 5:** For an assigned value of *w* and 100 initial estimates of the solution of Eq. (8), the solution with the least error in Eq. (8) and the corresponding errors in *T* and *V*.

Weight	Method	Best optimal solution									Statistics of ${\text{\% Error}}_{\text{obj}}$		
		Ar	H_2_	Current	PFR	$T$	$V$	${\%}{\text{Error}}_{ T}$	${\%}{\text{Error }}_{V}$	${\%}{\text{Error}}_{\text{obj}}$	Mean	Max	Min
0	Regression	66.93	6.54	302.56	0.3787	2556	160.95	14.81	0.59	0.59	6.91	11.13	0.59
	RSM	69.09	8.49	317.9	0.5318	2360	159.51	21.32	0.31	0.31	6.79	10.93	0.31
0.25	Regression	47.48	11.21	488.9	0.6	2963	168.87	1.25	5.54	4.47	6.05	8.06	4.47
	RSM	45.81	13.80	494.1	0.6	2978	166.78	0.74	4.24	3.36	5.72	8.10	3.36
0.5	Regression	47.93	9.26	484.5	0.6	2949	167.58	1.70	4.74	3.22	4.33	5.58	3.22
	RSM	44.33	15.00	506.7	0.6	3005	167.62	0.16	4.76	2.46	4.39	6.43	2.46
0.75	Regression	45.14	14.85	518.9	0.6	3015	169.83	0.48	6.14	1.90	2.66	3.65	1.90
	RSM	46.67	12.84	494.85	0.559	2977	169.33	0.75	5.83	2.02	2.57	3.15	2.02
1	Regression	46.58	11.90	501.6	0.364	2998	165.79	0.08	3.62	0.08	1.33	2.33	0.08
	RSM	47.48	11.52	511.2	0.424	2996	174.22	0.14	8.89	0.14	1.51	3.93	0.14

### Check if computed values of optimal input parameters give desired values of MPCs

**Figure 7:**
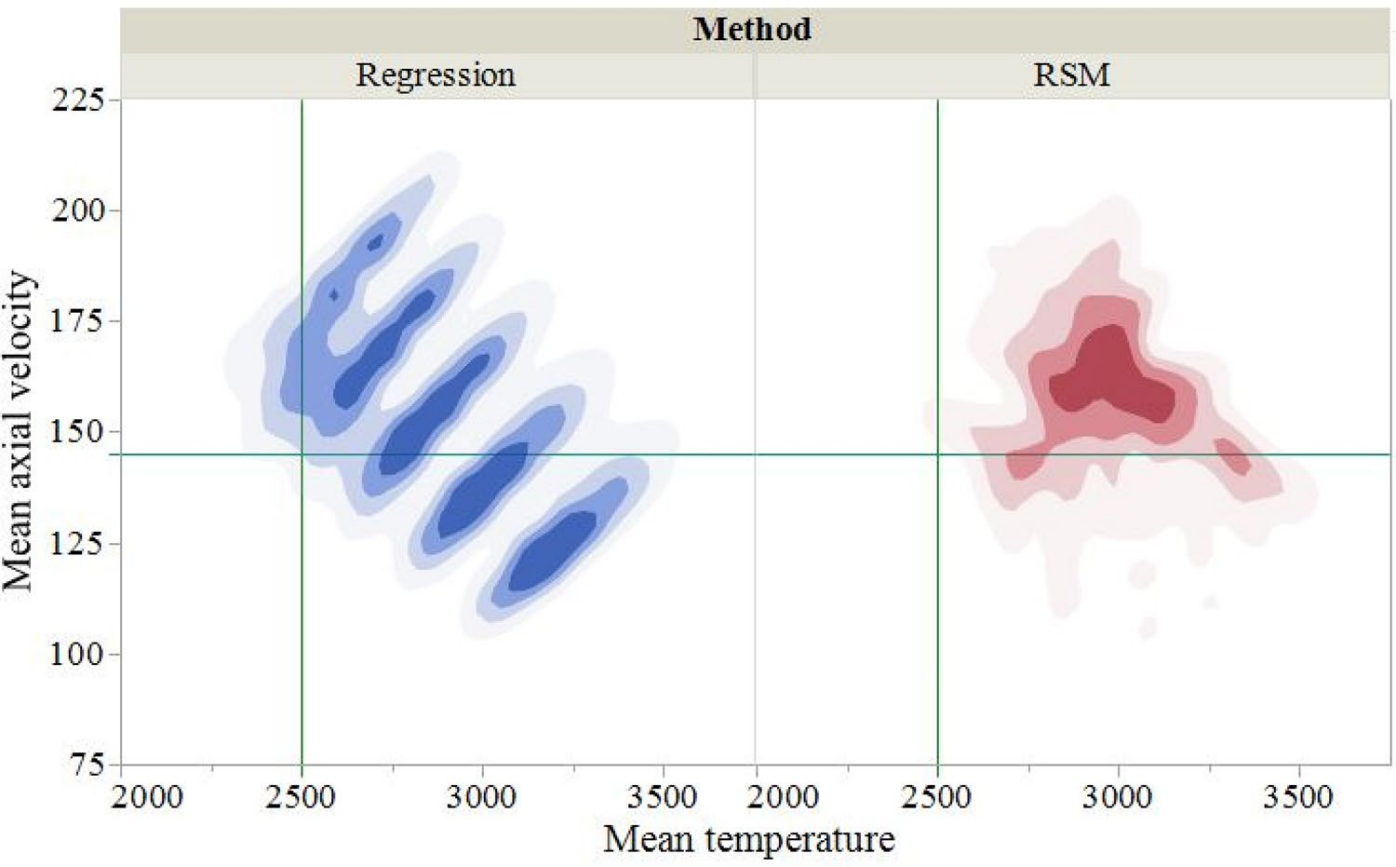
Contour plots of the data density for the RA and the RSM models from the 625 values of the input parameters. Note that the line T = 2500 K intersects the blue region corresponding to the RA but not the red region corresponding to the RSM.

As mentioned above the solution from an optimization algorithm depends upon the initial estimates of the solution. In order to show that the present approach provides values of input parameters for different desired values of MPCs that indeed will result in MPCs close to their desired values, we have considered four sets of desired values: (T_desired_, V_desired_) = (3000 K, 160 m/s), (2850 K, 180 m/s), (3200 K, 130 m/s) and (2500 K, 145 m/s). For each set, we considered 100 initial estimates to show how the corresponding solutions affect T_desired_ and V_desired_. In Table 6, for we have listed for $w = 0.5$ in Eq. (8) the best optimal input parameters, and the minimum and the maximum errors in the desired MPCs for these 100 solutions. Except for (T_desired_, V_desired_) = (2500 K, 145 m/s) and the RSM model, the predicted values of the input parameters give small errors in T_desired_ and V_desired_. In order to understand reasons for this, we have displayed in Fig. 7 contour plots of the MPCs predicted from the RA and the RSM using data for the 625 values of the input parameters in the range listed above. We can observe that there is no data density near (2500 K, 145 m/s) for the RSM model but there is for the RA model. Thus, these models are good only if T_desired_ and V_desired_ are within the range of outputs used to deduce them, i.e., these models cannot be extrapolated beyond the range of values of input parameters employed for their development since they are not physics-based. Similar remarks apply to ANN models.

**Table 6:** Optimal input parameters for the desirable MPCs with weighting coefficient, $w = 0.5$.

Desired values	Method	Best optimal solution									Statistics of $\% {\text{Error}}_{\text{obj}}$		
		Ar	H_2_	Current	PFR	$T$	$V$	${\%}{\text{Error}}_{ T}$	${\%}{\text{Error }}_{V}$	${\%} {\text{Error}}_ {\text{obj}}$	Mean	Max	Min
3000 K	Regression	47.93	9.26	484.5	0.5995	2949	167.6	1.70	4.74	3.22	4.33	5.58	3.22
160 m/s	RSM	44.33	15.00	506.7	0.6	3005	167.6	0.16	4.76	2.46	4.39	6.43	2.46
2850 K	Regression	59.04	3.13	567.8	0.5779	2870	189.9	0.72	5.49	3.10	4.91	6.60	3.10
180 m/s	RSM	60.97	10.85	515.8	0.4243	2814	182.5	1.26	1.40	1.33	4.44	7.17	1.33
3200 K	Regression	30.00	12.87	455.2	0.6	3164	134.2	1.13	3.23	2.18	3.39	7.26	2.18
130 m/s	RSM	30.79	5.85	373.8	0.2270	3160	130.1	1.25	0.09	0.67	2.61	11.75	0.67
2500 K	Regression	62.22	15.00	339.9	0.4142	2495	144.6	0.21	0.25	0.23	3.17	4.79	0.23
145 m/s	RSM	67.27	14.99	309.1	0.4698	2169	133.4	13.25	8.00	10.62	13.45	16.31	10.62

## Conclusions

The values of input parameters, namely, the argon flow rate, the hydrogen flow rate, the current, and the powder feed rate have been found to achieve the desirable mean particles’ temperature and velocity (i.e., mean particles’ characteristics, MPCs) in an atmospheric plasma spray process (APSP). The APSP has been numerically simulated by using the software LAVA-P-3D that has been shown by several investigators to predict the plasma flow and particle’s characteristics close to the corresponding experimental results. The analysis of variance (ANOVA) showed that the above mentioned four parameters significantly affect the MPCs. Subsequently, we used results from LAVA-P-3D for 625 numerical experiments in the regression analysis (RA) and the response surface methodology (RSM) to deduce MPCs as complete quadratic functions of the four input variables. Each method provided nonlinear algebraic equations for the mean particles’ velocity and temperature in terms of the four input parameters. For desired MPCs, these equations are solved for the four input parameters by using two optimization algorithms. Both the RA and the RSM predict values of input parameters that give MPCs within 5% of their desired values provided that they are within the range used to deduce the quadratic relations for the RA and the RSM.

We note that these methodologies can also be employed for ascertaining values of input parameters for desired outputs for other nonlinear complex processes.

## Supplementary information

Supplementary information.

## References

[1] Fauchais, P. L., Heberlein, Boulos, M. I. Overview of Thermal Spray. In *Thermal Spray Fundamentals*. (Springer, New York, 2014)

[2] Shang S, Guduri B, Cybulsky M, Batra RC (2014). Effect of turbulence modulation on three dimensional trajectories of powder particles in plasma spray process. J. Phys. D: Appl. Phys..

[3] Wan YP, Prasad V, Wang GX, Sampath S, Fincke JR (1999). Model and powder particle heating, melting, resolidification, and evaporation in plasma spraying processes. J. Heat Transfer.

[4] Zhang C (2009). Effect of in-flight particle characteristics on the coating properties of atmospheric plasma-sprayed 8 mol% Y2O3–ZrO2 electrolyte coating studying by artificial neural networks. Surf. Coatings Technol..

[5] Ramshaw J, Chang C (1992). Computational fluid dynamics modeling of multicomponent thermal plasmas. Plasma Chem. Plasma Process..

[6] Chang C, Ramshaw J (1994). Numerical simulation of nonequilibrium effects in an argon plasma jet. Phys. Plasmas..

[7] Wan Y, Prasad V, Wang GX, Sampath S, Fincke J (1999). Model and powder particle heating, melting, resolidification, and evaporation in plasma spraying processes. J. Heat Transfer..

[8] Wan Y (2001). Modeling and visualization of plasma spraying of functionally graded materials and its application to the optimization of spray conditions. J. Therm. Spray Technol..

[9] Xiong H-B, Zheng L-L, Sampath S, Williamson RL, Fincke JR (2004). Three-dimensional simulation of plasma spray: effects of carrier gas flow and particle injection on plasma jet and entrained particle behavior. Int. J. Heat Mass Transfer..

[10] Ramakrishnan S, Stokes AD, Lowke JJ (1978). An approximate model for high-current free-burning arcs. J. Phys D: Appl. Phys..

[11] Heimann RB (2010). Better quality control: stochastic approaches to optimize properties and performance of plasma-sprayed coatings. J. Therm. Spray Technol..

[12] Forghani SM, Ghazali MJ, Muchtar A, Daud AR (2014). Mechanical properties of plasma sprayed nanostructured TiO_2_ coatings on mild steel. Ceram. Int..

[13] Steinke T (2010). Process design and monitoring for plasma sprayed abradable coatings. J. Therm. Spray Technol..

[14] Muhammad MM, Isa MC, Shamsudin R, Jalar A (2014). Plasma spray deposition of fly ash onto mild steel substrates using a fractional factorial design approach. Ceram. Int..

[15] Schrijnemakers A, Francq BG, Cloots R, Vertruyen B, Boschini F (2013). Mullite plasma spraying for in situ repair of cracks in mullite refractories: simultaneous optimization of porosity and thickness by statistical design of experiments. J. Therm. Spray Technol..

[16] Lin B-T, Jean M-D, Chou J-H (2007). Using response surface methodology for optimizing deposited partially stabilized zirconia in plasma spraying. Appl. Surf. Sci..

[17] Gao F, Huang X, Liu R, Yang Q (2014). A study of pack aluminizing process for NiCrAlY coatings using response surface methodology. J. Mater. Eng. Perform..

[18] Liu K (2015). Particle in-flight behavior and its influence on the microstructure and mechanical property of plasma sprayed La2Ce2O7 thermal barrier coatings. Mater. Sci. Eng., A.

[19] Karthikeyan S, Balasubramanian V, Rajendran R (2014). Developing empirical relationships to estimate porosity and microhardness of plasma-sprayed YSZ coatings. Ceramics Int..

[20] Thirumalaikumarasamy D, Shanmugam K, Balasubramanian V (2012). Effect of atmospheric plasma spraying parameters on porosity level of alumina coatings. Surf. Eng..

[21] Pierlot C, Pawlowski L, Bigan M, Chagnon P (2008). Design of experiments in thermal spraying: a review. Surf. Coat. Technol..

[22] Guessasma S, Montavon G, Gougeon P, Coddet C (2003). Designing expert system using neural computation in view of the control of plasma spray processes. Mater. Des..

[23] Kanta A-F, Montavon G, Planche M-P, Coddet C (2009). Artificial neural networks implementation in plasma spray process: prediction of power parameters and in-flight particle characteristics vs. desired coating structural attributes. Surf. Coat. Technol..

[24] Kanta A-F, Planche M-P, Montavon G, Coddet C (2010). In-flight and upon impact particle characteristics modelling in plasma spray process. Surf. Coat. Technol..

[25] Choudhury TA, Hosseinzadeh N, Berndt CC (2011). Artificial Neural Network application for predicting in-flight particle characteristics of an atmospheric plasma spray process. Surf. Coat. Technol..

[26] Box GEP, Behnken DW (1960). Some new three level designs for the study of quantitative variables. Technometrics.

[27] Taetragool U, Sirinaovakul B, Achalakul T (2018). NeSS: an algorithm based on bees’ nest-site selection for combinatorial problems. Appl. Soft Comput..

[28] Goldberg DE (1989). Genetic Algorithms in Search, Optimization, and Machine Learning.

