# Supplementary material for: Numerical techniques to find optimal input parameters for achieving mean particles’ temperature and axial velocity in atmospheric plasma spray process
Source: Sci Rep. 2020 Dec 8;10:21483. doi: 10.1038/s41598-020-78424-w (PMC7722870; doi:10.1038/s41598-020-78424-w)
Supplement: Supplementary file 1 — Supplementary information. [file 41598_2020_78424_MOESM1_ESM.docx]

Supplementary Material

**Numerical Techniques to find Optimal Input Parameters for Achieving Mean Particles’ Temperature and Axial Velocity in Atmospheric Plasma Spray Process.**

R. C. Batra^1,^* and Unchalisa Taetragool^1,2 1^Department of Biomedical Engineering and Mechanics

Virginia Polytechnic Institute and State University Blacksburg, VA 24061

^2^ Department of Computer Engineering

King Mongkut's University of Technology Thonburi Bangkok 10140, Thailand

*Corresponding author; Email:rbatra@vt.edu; Tel. +1(540)231-6051

**Table S1** The ANOVA for three-factors *A*, *B* and *C* with respective levels *a*, *b* and *c*.

| **Source of variation** | **Degrees of freedom (DoF)** | **Sum of squares** | **Mean square** | **F-Value (F_0_)** |
| --- | --- | --- | --- | --- |
| $A$ | $a-1$ | $\mathrm{SS}_{A}$ | $\frac{\mathrm{SS}_{A}}{\mathrm{DoF}_{A}}$ | $\frac{\mathrm{SS}_{A}}{\mathrm{MS}_{E}}$ |
| $B$ | $b-1$ | $\mathrm{SS}_{B}$ | $\frac{\mathrm{SS}_{B}}{\mathrm{DoF}_{B}}$ | $\frac{\mathrm{SS}_{B}}{\mathrm{MS}_{E}}$ |
| $C$ | $c-1$ | $\mathrm{SS}_{C}$ | $\frac{\mathrm{SS}_{C}}{\mathrm{DoF}_{C}}$ | $\frac{\mathrm{SS}_{C}}{\mathrm{MS}_{E}}$ |
| $A*B$ | $\left( a-1 \right)\left( b-1 \right)$ | $\mathrm{SS}_{A,B}$ | $\frac{\mathrm{SS}_{A,B}}{\mathrm{DoF}_{A,B}}$ | $\frac{\mathrm{SS}_{A,B}}{\mathrm{MS}_{E}}$ |
| $A*C$ | $\left( a-1 \right)\left( c-1 \right)$ | $\mathrm{SS}_{A,C}$ | $\frac{\mathrm{SS}_{A,C}}{\mathrm{DoF}_{A,C}}$ | $\frac{\mathrm{SS}_{A,C}}{\mathrm{MS}_{E}}$ |
| $B*C$ | $\left( b-1 \right)\left( c-1 \right)$ | $\mathrm{SS}_{B,C}$ | $\frac{\mathrm{SS}_{B,C}}{\mathrm{DoF}_{B,C}}$ | $\frac{\mathrm{SS}_{B,C}}{\mathrm{MS}_{E}}$ |
| $\text{Error}$ | $\mathrm{DoF}_{E}$ | $\mathrm{SS}_{E}$ | $\frac{\mathrm{SS}_{E}}{\mathrm{DoF}_{E}}$ |  |
| $\text{Total}$ | $abc-1$ | $\mathrm{SS}_{T}$ |  |  |

Equation S1 is used to compute the degree of freedom of the error $(\mathrm{DoF}_{E}$), and equations for the other DoFs are listed in the second column of Table S1.

$\mathrm{DoF}_{E}=\mathrm{DoF}_{T}-\mathrm{DoF}_{A}-\mathrm{DoF}_{B}-\mathrm{DoF}_{C}-\mathrm{DoF}_{A,B}-\mathrm{DoF}_{A,C}-\mathrm{DoF}_{B,C}$ (S1)

Equations for the sum of squares listed in Table S1 are:

$\mathrm{SS}_{A}=\frac{1}{\mathrm{bc}}\sum_{i=1}^{a} y_{i..}^{2}-\frac{y_{\ldots}^{2}}{\mathrm{abc}}$, $y_{i..}=\sum_{j=1}^{b} \sum_{k=1}^{c} y_{\mathrm{ijk}}$, $y_{\ldots}=\sum_{i=1}^{a} \sum_{j=1}^{b} \sum_{k=1}^{c} y_{\mathrm{ijk}}$ (S2a, b, c)

$\mathrm{SS}_{A,B}=\frac{1}{c}\sum_{i=1}^{a} \sum_{j=1}^{b} y_{ij.}^{2}-\frac{y_{\ldots}^{2}}{\mathrm{abc}}-\mathrm{SS}_{A}-\mathrm{SS}_{B}$ (S3)

$y_{ij.}=\sum_{k=1}^{c} y_{\mathrm{ijk}}$ (S4)

$\mathrm{SS}_{T}=\sum_{i=1}^{a} \sum_{j=1}^{b} \sum_{k=1}^{c} y_{\mathrm{ijk}}^{2}-\frac{y_{\ldots}^{2}}{\mathrm{abc}}$ (S5)

$\mathrm{SS}_{E}=\mathrm{SS}_{T}-\mathrm{SS}_{A}-\mathrm{SS}_{B}-\mathrm{SS}_{C}-\mathrm{SS}_{A,B}-\mathrm{SS}_{A,C}-\mathrm{SS}_{B,C}$ (S6)

**Table S2** Four-Factor Box-Behnken Design

|  | **Actual Value** | | | | **Coded Value** | | | |
| --- | --- | --- | --- | --- | --- | --- | --- | --- |
|  | **Ar (slm)** | **H2**  **(slm)** | **Current (A)** | **PFR**  **(g/s)** | **Ar** | **H2** | **Current** | **PFR** |
| 1 | 30 | 3 | 450 | 0.4 | -1 | -1 | 0 | 0 |
| 2 | 30 | 9 | 300 | 0.4 | -1 | 0 | -1 | 0 |
| 3 | 30 | 9 | 450 | 0.2 | -1 | 0 | 0 | -1 |
| 4 | 30 | 9 | 450 | 0.6 | -1 | 0 | 0 | 1 |
| 5 | 30 | 9 | 600 | 0.4 | -1 | 0 | 1 | 0 |
| 6 | 30 | 15 | 450 | 0.4 | -1 | 1 | 0 | 0 |
| 7 | 50 | 3 | 300 | 0.4 | 0 | -1 | -1 | 0 |
| 8 | 50 | 3 | 450 | 0.2 | 0 | -1 | 0 | -1 |
| 9 | 50 | 3 | 450 | 0.6 | 0 | -1 | 0 | 1 |
| 10 | 50 | 3 | 600 | 0.4 | 0 | -1 | 1 | 0 |
| 11 | 50 | 9 | 300 | 0.2 | 0 | 1 | -1 | -1 |
| 12 | 50 | 9 | 300 | 0.6 | 0 | 0 | -1 | 1 |
| 13 | 50 | 9 | 450 | 0.4 | 0 | 0 | 0 | 0 |
| 14 | 50 | 9 | 450 | 0.4 | 0 | 0 | 0 | 0 |
| 15 | 50 | 9 | 450 | 0.4 | 0 | 0 | 0 | 0 |
| 16 | 50 | 9 | 600 | 0.2 | 0 | 0 | 1 | -1 |
| 17 | 50 | 9 | 600 | 0.6 | 0 | 0 | 1 | 1 |
| 18 | 50 | 15 | 300 | 0.4 | 0 | 1 | -1 | 0 |
| 19 | 50 | 15 | 450 | 0.2 | 0 | 1 | 0 | -1 |
| 20 | 50 | 15 | 450 | 0.6 | 0 | 1 | 0 | 1 |
| 21 | 50 | 15 | 600 | 0.4 | 0 | 1 | 1 | 0 |
| 22 | 70 | 3 | 450 | 0.4 | 1 | -1 | 0 | 0 |
| 23 | 70 | 9 | 300 | 0.4 | 1 | 0 | -1 | 0 |
| 24 | 70 | 9 | 450 | 0.2 | 1 | 0 | 0 | -1 |
| 25 | 70 | 9 | 450 | 0.6 | 1 | 0 | 0 | 1 |
| 26 | 70 | 9 | 600 | 0.4 | 1 | 0 | 1 | 0 |
| 27 | 70 | 15 | 450 | 0.4 | 1 | 1 | 0 | 0 |
